# Supplementary material for: Clinical, Histological and Genetic Characterisation of a Disorder of Sexual Development in a Pygmy Goat
Source: Animals (Basel). 2025 Mar 28;15(7):976. doi: 10.3390/ani15070976 (PMC11987831; doi:10.3390/ani15070976)
Supplement: Supplementary file 1 [file animals-15-00976-s001.zip › animals-3501150-supplementary.pdf]

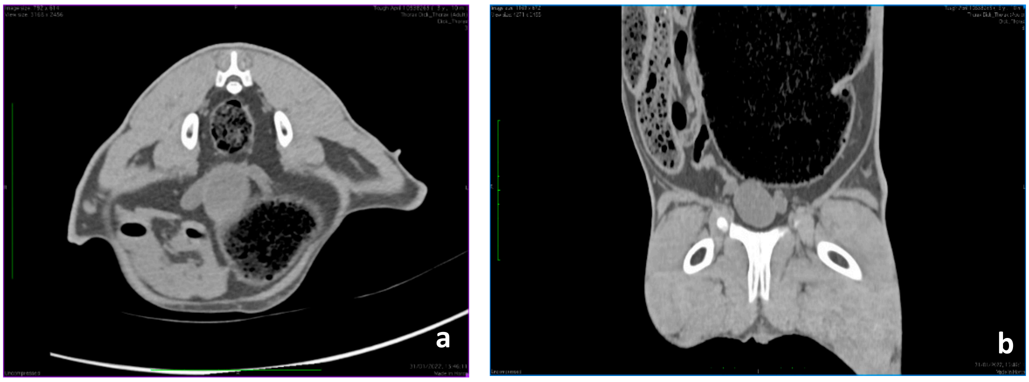

Figure S1. Computed tomography images. (a) Dorsal to the urinary bladder a fluid filled tubular structure is noted, which splits into a left and right branch at the level of the urinary bladder. This is consistent with a fluid filled uterus; (b) Dextrally, at the end of the uterine horn, there is a thin soft tissue band which travels laterodorsally. A similar structure can be seen sinistrally, however both end in bulbous soft tissue structures, which are located dextrally to the midline.

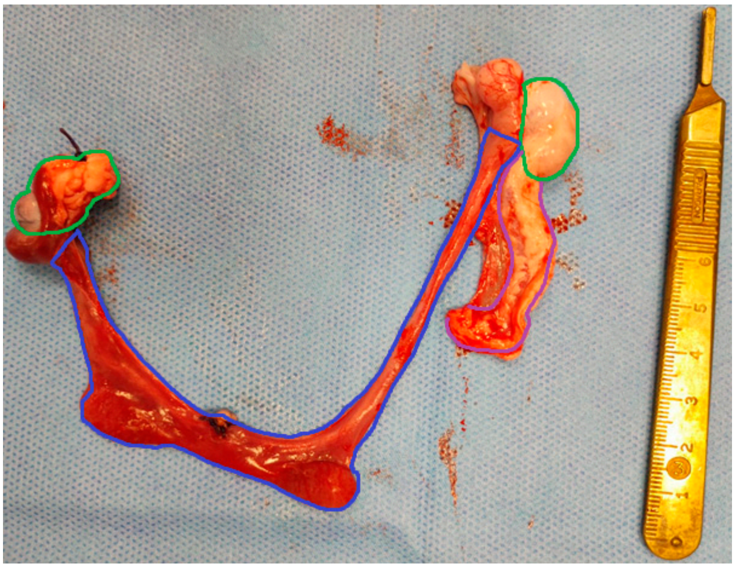

Figure S2. Gross morphology of the reproductive tract. Two small, tubular, bicornuated, filled-fluid structures resembling uterine horns (blue lines), thick-walled white tubular structure attached to the end of both horns resembling the vas deferens (purple lines), small rounded soft tissue masses resembling testis or ovotestis (green lines).

Table S1. Number of Illumina reads mapped to the Saanen_v.1 and ARS1.2 genomes for the patient (DSD Goat), Male control and Female Control.

Sample	Sex	*n* reads mapped to ARS1.2 (median coverage)	*n* reads mapped to Saanen_v.1 (median coverage)	*n* reads mapped to Saanen_v.1 after down-sampling (median coverage)
DSD Goat	Suspected DSD	237.8 (15X)	294.8 (15X)	NA
Male Control	Male	NA	358.7 (20X)	294.1 (16X)
Female Control	Female	NA	418.0 (22X)	292.6 (15X)

Table S2. Read mapping statistics for the regions chr10, chrX and chrY

Sample	Sex	chr10 (median coverage)	chrX (median coverage)	*n* chrY (median coverage)	*X:Y ratio*	*X:chr10 ratio*	*Y:chr10 ratio*
DSD Goat	Suspected DSD	11.4M (15X)	13.5M (12X)	0.3M (3X)	4	0.8	0.2
Male Control	Male	11.8M (16X)	8.9M (8X)	0.8M (8X)	1	0.5	0.5
Female Control	Female	11.5 (15X)	15.4M (14X)	0	NA	0.93	NA
